# The effect of curriculum sample selection for medical school

**DOI:** 10.1007/s10459-016-9681-x

**Published:** 2016-04-23

**Authors:** Marieke de Visser, Cornelia Fluit, Jaap Fransen, Mieke Latijnhouwers, Janke Cohen-Schotanus, Roland Laan

**Affiliations:** 1 Radboudumc Health Academy, Research of Learning and Education, Radboud University Medical Center, Huispost 42, Postbus 9101, 6500 HB Nijmegen, The Netherlands; 2 Department of Rheumatology, Radboud University Medical Center, Nijmegen, The Netherlands; 3 Center for Research and Innovation in Medical Education, University of Groningen and University Medical Center Groningen, University of Groningen, Groningen, The Netherlands

**Keywords:** Academic performance, Selection, Undergraduate medical education

## Abstract

In the Netherlands, students are admitted to medical school through (1) selection, (2) direct access by high pre-university Grade Point Average (pu-GPA), (3) lottery after being rejected in the selection procedure, or (4) lottery. At Radboud University Medical Center, 2010 was the first year we selected applicants. We designed a procedure based on tasks mimicking the reality of early medical school. Applicants took an online course followed by an on-site exam, resembling courses and exams in early medical school. Based on the exam scores, applicants were selected or rejected. The aim of our study is to determine whether curriculum sample selection explains performance in medical school and is preferable compared to selection based on performance in secondary school. We gathered data on the performance of students of three consecutive cohorts (2010–2012, N = 954). We compared medical school performance (course credits and grade points) of selected students to the three groups admitted in other ways, especially lottery admissions. In regression analyses, we controlled for out of context cognitive performance by adjusting for pu-GPA. Selection-admitted students outperformed lottery-admitted students on most outcome measures, unadjusted as well as adjusted for pu-GPA (*p* ≤ 0.05). They had higher grade points than non-selected lottery students, both unadjusted and adjusted for pu-GPA (*p* ≤ 0.025). Adjusted for pu-GPA, selection-admitted students and high-pu-GPA students performed equally. We recommend this selection procedure as it adds to secondary school cognitive performance for the general population of students, is efficient for large numbers of applicants and not labour-intensive.

## Introduction

Worldwide, there are more applicants for medical school than capacity available. Medical schools adopt a variety of procedures to select their intended student population out of many seemingly suitable applicants. Overview studies show that prior cognitive achievement is an important predictor for achievement in medical school, especially during the early years (Ferguson et al. 2002; Siu and Reiter 2009). In selection practice, prior cognitive achievement is often defined by pre-university Grade Point Average (pu-GPA). However, pu-GPA represents *overall* cognitive performance in several pre-university subjects and medical schools aim to forecast performance in the *specific* domain of medical education. Selection based on cognitive performance resembling this specific ability could predict performance in medical school better than overall cognitive performance. Consequently, applicants could be selected or rejected incorrectly, if selection is not based on specific performance representing the curriculum they apply for. In a review study on admission, Kuncel and Hezlett (2007) postulate that, in graduate school selection, most effective predictors for success are directly connected to the discipline involved. More specifically, in their recommendations for selection Prideaux et al. (2011) emphasize that selection should be aligned with the programme that is selected for (i.e. ‘developing congruity between selection, curriculum and assessment’). A selection procedure based on work sample testing could be a way to combine these perspectives (Ployhart et al. 2006; Meijer and Niessen 2015). Work sample testing is described and studied extensively in personnel selection literature and focuses on situation specific performance. It can be defined as ‘a test in which the applicant performs a selected set of actual tasks that are similar to those performed on the job’ (Ployhart et al. 2006). Also, fidelity between the exam and the nature of the actual tasks to be done afterwards, is an important mechanism in the predictive validity of these tests (Guion 1998).

We therefore designed a selection procedure in which applicants are tested on tasks that resemble those in early medical school (‘the job’) as much as possible. In this study we call this approach a curriculum sample selection. The procedure aims to select for the first year of medical school as from our point of view year 1 itself selects for the subsequent years and the curriculum as a whole prepares students to be good doctors. Accordingly, selection for medical school is not selection of the best doctors, but should be based on the applicant’s capability of being successful in medical school.

In the Netherlands, students choose a specific programme as soon as they start undergraduate education. The study of medicine involves a 6 years programme that follows directly after graduation from secondary school, mostly at the age of 18. Three different routes of admission are applicable as described in more detail by Schripsema et al. (2014): (1) direct access through excellent secondary school performance (high pu-GPA), (2) selection and (3) a lottery procedure, which includes admission of non-selected applicants. Consequently, each cohort of students starting medical school consists of students admitted through different routes, which allows us to compare groups of students within the same cohort at one medical school. This contrasts with most international studies, which include students admitted by just one procedure per cohort. Selection is voluntary for applicants, and each Dutch medical school employs its own procedure and defines the percentage of students admitted by selection (up to 50 % in the timeframe of our study). Besides the general predictive value of pu-GPA mentioned above, research in Dutch context has already shown that students admitted directly through high pu-GPA outperform students admitted otherwise (Schripsema et al. 2014). However, this is only a small subgroup (approximately 5 % of the students finishing the highest secondary school level in 2012) (Ministry of Education 2015), and there is much more capacity in medical education than high pu-GPA students applying. Furthermore, the law concerning admittance to higher education in the Netherlands has changed and the system of direct access through high pu-GPA will end (Ministry of Education 2014). Therefore, our primary interest in this study is the major population of applicants, who do not perform excellently at secondary school.

The aim of our study is to determine whether curriculum sample selection (1) explains performance in medical school and (2) is preferable compared to selection based on performance in secondary school. Our research question is: “Do curriculum sample selected students perform differently compared to students admitted otherwise regarding results in the first 3 years of medical school, taking into account secondary school performance defined by pu-GPA?”

## Methods

### Setting

**Fig. 1:**
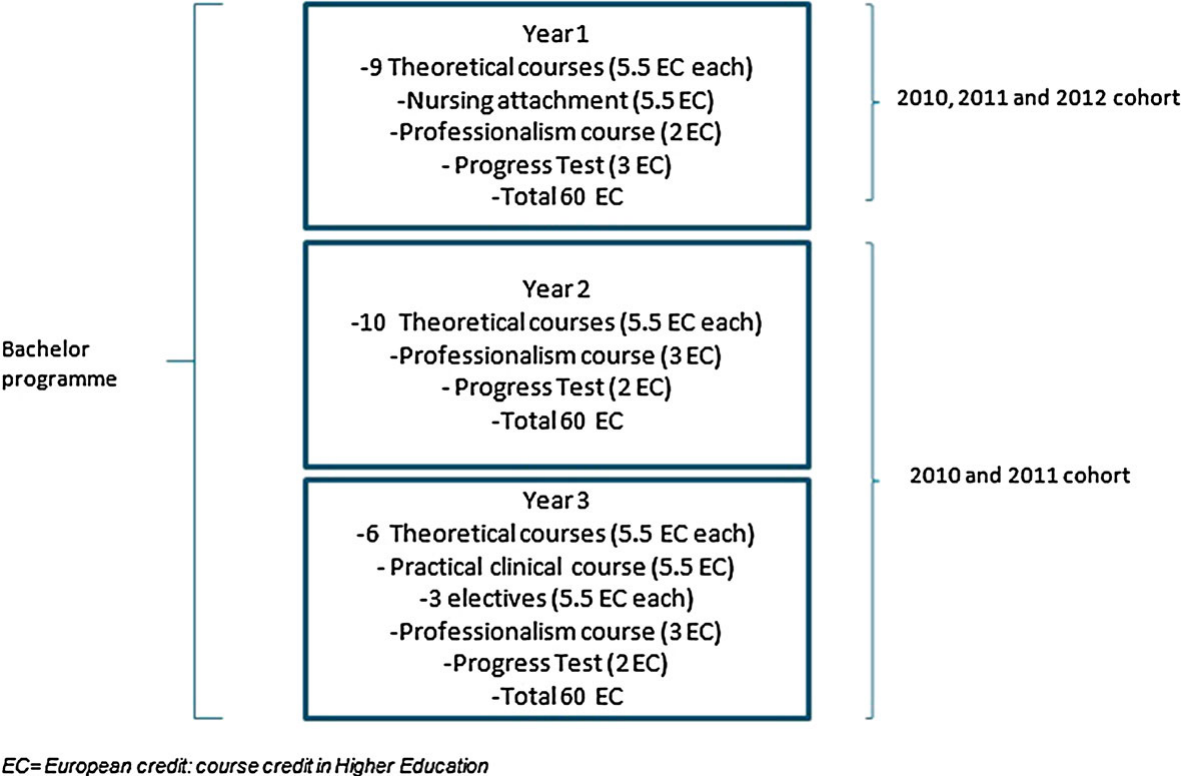
RUMC Bachelor’s Programme

This study was performed at the Radboud University Medical Center in Nijmegen, the Netherlands (RUMC). In Dutch medical education, a 3-year mainly theoretical Bachelor’s programme (Fig. 1) is followed by a 3-year Master’s programme with mainly practical education. Nevertheless, two courses in the RUMC Bachelor’s programme focus on practical training. In the first year nursing attachment students work in a nursery home (Helmich et al. 2012). In the third year practical clinical course, they are introduced to history taking, physical examination and clinical reasoning. Each year, 330 new students are admitted.

In the timeframe of our study, applicants could choose to participate in selection. If they did not, they automatically participated in the national lottery procedure. In 2010 and 2011, half the capacity of 330 was available for selection admissions and high pu-GPA admissions. In 2012, half the capacity of 330 was available for selection admissions only and the other half for lottery and high pu-GPA admissions. The first year we selected students was 2010. Before then, only lottery admissions and high pu-GPA admissions were applicable.

### Population

A total of 954 students who enrolled in their Bachelor’s programme in medical education at the RUMC in September 2010, 2011 or 2012 were included in the study (Table 1). Students whose data were incomplete or who enrolled in an individual track because of relevant prior education at a university level, were excluded from the study (n = 36).

**Table 1:** Descriptive statistics cohorts 2010–2012

	Selection admissions (1)	High pu-GPA admissions (2)	Non-selected lottery admissions (3)	Lottery admissions (4)	All admissions
n	374	163	137	280	954
% Female	70	71	57**	63	66
Age (SD), years	18.5 (.63)	18.5 (1.1)	18.5 (.67)	19.7 (2.3)*	18.9 (1.5)
pu-GPA (SD)	7.0 (.49)	8.0 (.36)**	6.8 (.54)**	6.7 (.63)*	7.1 (.69)

pu-GPA is composed of pre-university grades in Dutch, English, Biology, Physics, Chemistry (the only subjects all students had in common)

Refence group: selection admissions (1)

* Significant difference at a *p* ≤ 0.05 level

** Significant difference at a *p* ≤ 0.025 level

### Three admission routes

- High pu-GPA

In the Netherlands, students have direct access to medical school if their pu-GPA is equal to or higher than 8 on a scale of 1 (poor)–10 (excellent). Compulsory subjects included are Dutch, English, Biology, Physics and Chemistry. Mathematics is a compulsory subject as well but is offered in different variations. Other subjects depend on students’ personal choices.

- Lottery procedure

According to Dutch law, lottery applicants are classified into four categories depending on their pu-GPA, and lots are drawn within each category (7.9–7.5, 7.4–7.0, 6.9–6.5, 6.4–6.0) in a 9:6:4:3 ratio.

- RUMC selection

Based on the evidence of the predictive value of prior cognitive achievement and of work sample exams, we designed a selection procedure that mimicked the first part of early medical school. It consisted of an online course followed by an exam. The course and the exam were designed to mimic the courses and examinations in our programme as closely as possible, given the restraints of an online learning environment. Because the selection procedure resembled the content, required learning strategies and assessment procedures at our medical school, we assumed that scores in our exams would be a reliable predictor of success in the first 3 years of study. As the procedure took place when students were preparing for their pre-university exams, we assumed that motivation and planning skills were indirectly measured besides specific cognitive skills.

The selection procedure was open to all applicants who were about to finish secondary school. Once admitted to the selection procedure, applicants were enrolled in a course in the digital learning platform used by the RUMC. In 2010, the course topic was cervical cancer and human papilloma virus (HPV), in 2011 it was rheumatic disorders, and in 2012 it was the ageing brain. Basic biomedical, clinical, sociological, ethical and psychological perspectives were integrated into the course, like in the medical school curriculum. Applicants took the online course at home during 4 weeks. The estimated course load was 80 h. The course comprised lectures, assignments and forums, simulating real medical education in Nijmegen. Teachers moderated the forums to some extent and corrected apparent misconceptions.

After their preparation period, applicants took an on-site multiple choice test (70 % weight in final score) and wrote an essay focusing on psychological, ethical and social aspects of the study subject (30 %). Besides content aspects, essays were also assessed on structure, language and writing style. The test was taken by 392 applicants in 2010, 426 in 2011 and 441 in 2012, and, after final scores had been ranked, 106, 104 and 164 applicants were admitted, respectively. Rejected applicants automatically participated in the lottery procedure.

Four categories of students are distinguished in this study: (1) selected admissions: students admitted through our curriculum sample selection procedure; (2) high pu-GPA admissions: students admitted because of excellent performance in secondary school; (3) non-selected lottery admissions: students who had been rejected in the selection procedure and were subsequently admitted through the national lottery procedure; (4) lottery admissions: students who had not participated in the selection procedure and were admitted through the national lottery procedure.

### Measures

The primary outcome measure was the percentage of students obtaining ≥42 out of the compulsory 60 first-year credits. This is an important threshold, as from 2011 onwards, students obtaining fewer than 42 credits in year 1 have to leave medical school.

Additionally, we used measures (credits and grades) for different types of performance (theoretical and practical) in year 1 (all cohorts) and in year 2 plus 3 (cohorts 2010 en 2011; cohort 2012 had not finished 3 years at the time frame of our study). For grades, we only counted a student’s first examination attempt.

Secondary outcome measures:

1. Drop-out percentages: the combination of the percentage of students not obtaining ≥42 credits (primary outcome measure, involuntary withdrawal) and voluntary withdrawal;
2. Percentage of students receiving all 60 credits in year 1;
3. Average grade point for theoretical exams in year 1 (scale: 1 (poor)–10 (excellent)), excluding students who took fewer than two out of nine exams (n = 9);
4. Percentage of students obtaining their Bachelor’s degrees within 3 years of study;
5. Average grade point in 2nd and 3rd year theoretical exams, excluding students who took fewer than four out of sixteen exams (n = 7);
6. Average grade point for practical clinical course in year 3;
7. Percentage of students receiving the maximum grade for the first-year nursing attachment (scale: insufficient-sufficient-good);

Regarding the Bachelor’s results of the 2010 and 2011 cohorts, only students who had obtained ≥42 credits in their first year were included (n = 570; excluded from 2010 cohort: 26; 2011 cohort: 30). This concerns outcome measures 4, 5, and 6.

### Data collection

pu-GPA data of the five compulsory subjects were made available by the Ministry of Education. All other data were collected from the RUMC student administration. Our institute waived approval and by Dutch law, no ethical approval is applicable to studies like ours, using regularly registered data. Data were treated strictly confidentially and were available for the researchers only. All analyses were conducted anonymously.

### Data analysis

The main analyses of interest are the differences in primary and secondary outcome measures between admission categories. First of all, we tested for all of the outcome measures if admission categories differed, using χ^2^ tests (categorical variables), or ANOVA (continuous variables). As selection was new at our medical school when we launched our study, we wanted to compare selection admissions to other admissions routes. We chose selection admissions as our reference group and lottery admissions as the planned primary contrast. To analyze whether there was a difference in proportion of students obtaining ≥42 first-year credits (primary outcome) between the admission categories, logistic regression was used. To control for secondary school performance, we adjusted for pu-GPA. This was computed as a mean score for the five subjects that all students had in common. We additionally controlled for sex, age and cohort if their addition to the regression model influenced the effect (regression coefficients) of the independent variable ‘admission route’ for more than 10 % (Grobbee and Hoes 2009). The analyses for the descriptive data and the secondary outcome measures except drop-out were performed similarly, using logistic or linear regression, as appropriate. For the planned contrast, we used α = .05. For the other two contrasts (selection admissions compared to high pu-GPA admissions and non-selected lottery admissions), we used α/2 = .025 to correct for multiple comparisons according to the Bonferroni method (Petrie and Sabin 2009). Post hoc, for drop-out we performed χ^2^ tests to find out which groups differed.

Post hoc we used the primary outcome measure and the drop-out measure to explore the additional value of our selection procedure for different pu-GPA categories graphically. We included our planned primary contrast and added high pu-GPA admissions as a benchmark. We created these categories according to the lottery system categories, based on the data available (GPA of five compulsory subjects).

The statistical package for social sciences (SPSS) Windows version 20 was used for the statistical analyses.

## Results

### Descriptives

Descriptive statistics of the cohorts 2010–2012 categorized by admission route are shown in Table 1. Compared to the group of non-selected lottery admissions, the group of selected students included a higher percentage of females. Compared to the lottery admitted students, the group of selected students had a lower mean age. The group of selected students had a higher pu-GPA than both non-selected lottery admissions and lottery-admitted students and a lower pu-GPA compared to pu-GPA admitted students. Pu-GPA of the five subjects all students had in common was available for most of the population (n = 868): data were missing for 7 % of selection admissions, 6 % of high pu-GPA admissions, 4 % of non-selected lottery admissions and 17 % of lottery admissions. There were no other missing outcomes or covariates.

### ≥42 credits year 1

Univariately, the percentage of students receiving ≥42 credits in year 1 differed among groups ($\chi_{(3)}^{2} = 32.92$, *p* ≤ 0.001). Compared to selected students, a lower percentage of the lottery-admitted students received ≥42 credits (96 vs 86, *p* = .001), adjusted for pu-GPA and age as well (*p* = .03). No differences were found between selected students and high pu-GPA admitted students and non-selected lottery admissions (Table 2).

**Table 2:** Course credits year 1 and Bachelor’s degree within 3 years

	N	% of students^c^	β	*p*	OR
≥*42 credits year 1* ^a^					
Selected	374 (349)	96	Ref	Ref	Ref
High pu-GPA admission	162 (153)	99	1.27 (.01)	.09 (.99)	3.6 (1.0)
Non-selected lottery	135 (130)	93	−.58 (−.30)	.16 (.49)	.56 (.74)
Lottery	263 (222)	86	−1.30 (−.77)	.00^1^ (.03^1^)	.27 (.47)
*60 credits year 1* ^b^					
Selected	374 (349)	74	Ref	Ref	Ref
High pu-GPA admission	162 (153)	90	1.19 (.06)	.00^2^ (.85)	3.29 (1.06)
Non-selected lottery	135 (130)	59	−.65 (−.49)	.00^2^ (.03)	.52 (.61)
Lottery	263 (222)	56	−.80 (−.61)	.00^1^ (.00^1^)	.45 (.55)
*Bachelor’s degree within 3* *years* ^b^					
Selected	203 (198)	79	Ref	Ref	Ref
High pu-GPA admission	109 (109)	81	.11 (−.68)	.70 (.06)	1.12 (.51)
Non-selected lottery	92 (91)	64	−.74 (−.56)	.01^2^ (.05)	.48 (.57)
Lottery	166 (141)	66	−.66 (−.32)	.01^1^ (.22)	.52 (.72)

N in parentheses lower because of pu-GPA missing data

1 = Significant difference at a *p* ≤ 0.05 level, 2 = significant difference at a *p* ≤ 0.025 level

^a^In parentheses: adjusted for pu-GPA and age, which were the only confounders

^b^In parentheses: adjusted for pu-GPA, no other confounders applicable

^c^Percentage of students obtaining ≥42, 60 credits year 1, Bachelor’s degree within 3 years respectively

### Dropout

The total drop-out percentage is 8.9. The percentages differed among groups ($\chi_{(3)}^{2} = 57.07$, *p* ≤ 0.001). Compared to the selected group, only the lottery admitted group showed a significant difference (*p* = .001) (selected: 4.3 %; high pu-GPA admission: 1.8 %; non-selected lottery admissions: 8,8 %; lottery admission: 19.3 %).

## 60 credits year 1

The percentage of students receiving all 60 credits within 1 year differed among groups ($\chi_{(3)}^{2} = 66.02$, *p* ≤ 0.001). Compared to selected students, a lower percentage of lottery-admitted students obtained all 60 credits in 1 year (74 vs 56, *p* = 0.001), unadjusted and adjusted for pu-GPA as well (*p* = .001). Compared to selected students, a higher percentage (90) of high pu-GPA admitted students received 60 credits, and a lower percentage (59) of non-selected lottery admissions did. Adjusted for pu-GPA, no differences remained (Table 2).

### Bachelor’s degree within 3 years of study

Within the group of students receiving ≥42 credits in their first year, the percentage of students obtaining their Bachelor’s degree within 3 years differed among groups ($\chi_{(3)}^{2} = 14.91$, *p* ≤ 0.05). Compared to selected students, a lower percentage of lottery-admitted students obtained their Bachelor’s degree within 3 years of study (79 vs 66, *p* = .01). Adjusted for pu-GPA, no difference remained (*p* = .22). No difference was found with high pu-GPA admitted students; adjusted for pu-GPA, no difference with non-selected lottery admissions was found (Table 2).

### Average grade point year 1 and average grade point years 2–3

The average grade point on exams in year 1 differed between groups F_(3,936)_ = 102.60, *p* ≤ 0.001. Selected students had a higher GPA in year one than lottery-admitted students (6.9 vs 6.5, *p* = .001). The average grade point on exams in year 2 plus 3 differed between groups F_(3,559)_ = 37.93, *p* ≤ 0.001. Lottery-admitted students had a lower average grade point in year 2 and 3 than selected students (7.0 vs 6.7, *p* = .001). Adjusted for pu-GPA, no differences remained (*p* = .12). In both year 1 and year 2 plus 3, selected students outperformed non-selected lottery admissions; unadjusted for pu-GPA, high pu-GPA admitted students outperformed selected students (Table 3).

**Table 3:** Average grade points in theoretical exams

	N	GPA (SD)	β	T	*p*
*Average grade point year 1* ^*a*^					
Selected	373 (348)	6.9;.76	Ref	Ref	Ref
High pu-GPA admission	163 (154)	7.8; .71	.91 (.07)	11.46 (.83)	.00^2^ (.41)
Non-selected lottery	136 (131)	6.5; .87	−.44 (−.27)	−5.18 (−3.50)	.00^2^ (.00^2^)
Lottery	268 (222)	6.5; 1.0	−.48 (−.26)	−7.02 (−4.04)	.00^1^ (.00^1^)
*Average grade point year 2*–*3* ^*a*^					
Selected	202 (197)	7.0; .67	Ref	Ref	Ref
High pu-GPA admission	105 (105)	7.5; .70	.54 (.07)	6.37 (.71)	.00^2^ (.48)
Non-selected lottery	92 (91)	6.6; .77	−.39 (−.25)	−4.36(−2.29)	.00^2^ (.00^2^)
Lottery	164 (139)	6.7;.73	−.29 (−.12)	−3.85(−1.57)	.00^1^ (.12)

N in parentheses lower because of pu-GPA missing data

^a^In parentheses: adjusted for pu-GPA, no other confounders applicable

1 = Significant difference at a *p* ≤ 0.05 level, 2 = significant difference at a *p* ≤ 0.025 level

### Nursing attachment

The percentage of students gaining the highest score for the first-year nursing attachment (selected: 77 %, high pu-GPA admission: 82 %, non-selected lottery: 79 %, lottery: 78 %) did not differ among groups ($\chi_{(3)}^{2} = 1.88$, *p* > .05).

### Practical clinical course

The grade point for a practical clinical course differed between groups F_(3,527)_ = 3.42, *p* ≤ 0.05. Selected students had a higher grade point than lottery-admitted students (6.9 vs 6.7, *p* = .001), adjusted for pu-GPA as well. Selected students had a lower grade point than high pu-GPA admitted students (6.9 vs 7.2, *p* = .01). Adjusted for pu-GPA, no significant differences remained. *Detailed data not shown.*

### Pu-GPA and effects of selection

**Fig. 2:**
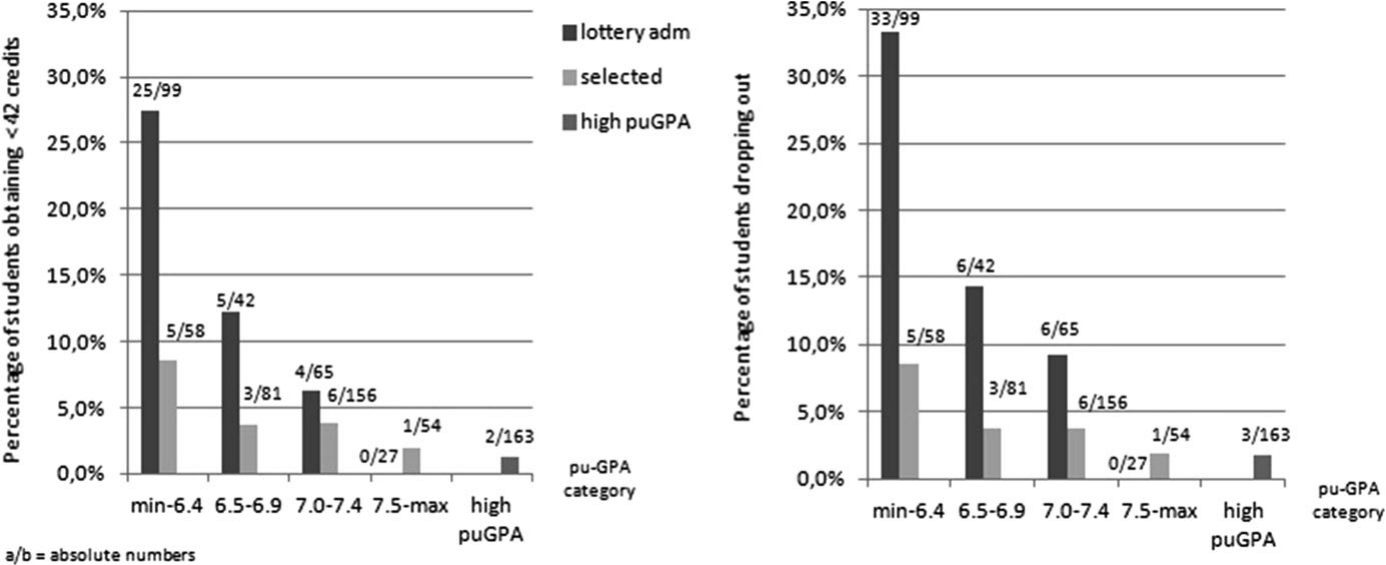
Drop-out and <42 credits, per pu-GPA category

The post hoc graphical analyses indicate a stronger additional effect of selection compared to pu-GPA for the lower pu-GPA categories regarding the primary outcome measure and drop-out (Fig. 2).

## Discussion

For the general population of students we focus on, our curriculum sample selection procedure shows additional value compared to secondary school cognitive achievement (pu-GPA). According to the results of this comparative study, selected students outperform lottery-admitted students who did not participate in the selection procedure. Adjusted for pu-GPA, differences between selected and lottery-admitted students remain significant for most of the outcome measures. The additional value of our selection procedure seems the strongest in the lower pu-GPA-categories.

In year 1, selected students more often obtain the necessary minimum of 42 and the maximum of 60 credits, do not withdraw voluntarily and obtain higher grades in theoretical exams. In years 2 and 3, the differences between the groups decrease as we only included students who obtained at least 42 credits in year 1. While our curriculum sample selection procedure selects for year 1, year 1 itself selects for the subsequent years of medical school. Nevertheless, the percentage of students obtaining their Bachelor’s degree within 3 years is higher for selected students than for lottery-admitted students. A second explanation for the decreasing effect of the admission route over the years may be that the curriculum may influence students’ learning patterns (Van der Veken et al. 2009; Bitran et al. 2012). We assume that, over time, students know more clearly what is expected of them and what learning strategy they need to pass their exams, and that they adjust their strategy accordingly (Miller 1990), which makes the groups of students more alike.

Regarding grades, the differences in year 1 and year 2–3 are relatively small (however significant). It’s influence on clinical practice is yet unclear. Selected students have higher grade points than non-selected lottery admissions during the Bachelor’s programme. On a group level, therefore, we seem to select and reject appropriately, although adjusted for pu-GPA no differences in credits gained were found.

Lastly, high pu-GPA admitted students outperform or perform equal to selected students, as we expected based on previous research (Schripsema et al. 2014). The high pu-GPA threshold of 8 is a threshold by law. However, we wondered which threshold would be applicable based on our selection practice data. The data indicate that the high pu-GPA threshold can possibly be lowered to 7.5, as the drop-out rate would not rise. Nevertheless, if the high pu-GPA threshold would be 7.5, selection is still necessary as only around 25 % of our population has a pu-GPA of 7.5 or above. Also, high pu-GPA can only be used as a selection criterion, if it is comparable between applicants. Unlike in many other countries, in the Dutch educational system pu-GPA is uniformly composed and registered and, therefore, comparable nationwide. This provides a reliable measure for secondary school performance for all of our applicants.

Overall, we found that the higher the applicants’ pu-GPA, the lower the additional value of our selection procedure regarding the percentage of students obtaining <42 credits and drop-out rates. Our graphs indicate that the curriculum sample selected students with lower pu-GPA’s perform almost equally compared to the selected students in the higher pu-GPA categories.

How do our findings compare to other studies? In general, cognition based selection procedures seem to predict success in the early years of study (Ferguson et al. 2002; Siu and Reiter 2009). Our curriculum sample selection is a cognitive approach as well and adds to this previous research. Also non-cognitive tests like the multiple mini-interview have shown predictive validity for future performance (Reiter et al. 2007; Siu and Reiter 2009). In most selection studies, no control groups have been used. In the Dutch situation control groups are available. Our findings are in line with a recent study on a Dutch medical school in Groningen (Schripsema et al. 2014) studying a very different selection procedure, including cognitive and non-cognitive elements. Urlings-Strop et al. (2013) also found that selected students outperform non-selected students concerning clerkship GPA and drop-out (Erasmus MC Rotterdam). Selected students appear to perform better in medical school than lottery-admitted students, therefore, independent of the type of selection procedure.

### Possible explanations

Next to the effect of self-selection studied by Urlings-Strop et al. (2013), an explanation for the effect of selection in general can be that being selected raises the students’ self-efficacy and thus stimulates performance (Bandura 1977). The feeling of outperforming others during selection may be a strong mechanism for good subsequent performance.

Based on the evidence referred to in the introduction of this study (Guion 1998; Ferguson et al. 2002; Ployhart et al. 2006; Siu and Reiter 2009; Meijer and Niessen 2015), we think that curriculum sample selected students outperform lottery admitted students because our selection procedure requires applicants to perform in a situation similar to the real world of early medical school. They have shown they perform well on authentic tasks during the selection procedure, representing what needs to be done in early medical school (Koens et al. 2005; Patterson et al. 2008). Another perspective is that the selection procedure might be a learning tool itself (assessment *as* learning) for the participants who succeeded as it may help them to acquire job knowledge that is relevant for medical school. Job knowledge has originally been defined as ‘knowing what to do and how to do it’ (McCloy et al. 1994). In a review study, Kuncel et al. link ‘job knowledge’ to education, interpreting ‘job’ as graduate school. They state that “one would expect that a student entering with more ‘job’ knowledge would perform better than one who has less ‘job’ knowledge. The students with greater job knowledge would have a better framework to integrate field-specific knowledge, enhancing learning.” (Kuncel et al. 2001). Although we study selection for undergraduate education, this mechanism could be applicable here as well.

How can we explain that selected students perform better than would be expected based on their pu-GPA and perform equally compared to high pu-GPA admitted students? Possibly, the former perform better in medical school setting compared to what could be expected based on pre-university results solely, because they experience positive affect. Positive affect is a predictor of student performance (Fredrickson 2001; Gillet et al. 2013). It “reflects the extent to which a person feels enthusiastic, active and alert.”(Watson et al. 1988). This may be of less influence for the high pu-GPA admitted group because of a ceiling effect. This positive affect may be caused by the context of medical school. Research indicates that cognitive skills are context-specific (Perkins and Salomon 1989; Eva et al. 1998), although a scattered picture arises from different studies. Koens et al. (2005) aim to disentangle the diffuse concept of context in medical education and developed a three dimensional model. The dimensions are the physical, the semantic and the commitment dimension. The commitment dimension in particular may partly explain the effect of our selection procedure: the medical school setting of the procedure could generate more applicant commitment than the pre-university setting, encouraging applicants to perform beyond expectations based on their pu-GPA.

### Strengths and limitations

Our study’s follow-up is relatively short. It is as yet unclear how students perform in their practically oriented Master’s programme. However, no differences were found in the nursing attachment. The possible concern that students selected mainly on their performance in cognitive tasks similar to those in early medical school would underperform in a practical medical setting seems to be unwarranted based on these data. Another limitation of our study is that, in 2010, the university’s end-of-first-year assessment did not yet have any formal consequences. Therefore, this is an external mechanism possibly influencing our results. Nevertheless, no cohort effects were found. Furthermore, this study is limited by the fact that percentages of pu-GPA missing data were unequal between the lottery-admitted and the three other groups. The strength of our study is that it explores a new selection method, based on strong similarities with early medical school process and content. It offers the opportunity to compare groups within one medical school and is combining three consecutive cohorts.

### Implications for practice

Our outcomes are the result of selection by a curriculum-based part-time online course taking only 1 month. Such a selection procedure is relatively achievable, even for high numbers of applicants, compared to the available capacity. The yearly costs for carrying out the selection procedure at our institute are approximately €60.000. In 2010–2012, each year an average of 600 applicants signed in for the selection procedure, so costs are around €100 per applicant per year. Course content can be taken from the regular curriculum, and the procedure is accessible without previous exams or tests. However, we can not rule out the possibility that our selection procedure is subject to socio-cultural inequality through coaching effects.

### Further research

In this study, applicants were selected on a cognitive basis though non-cognitive skills as well are important both in medical school and in practice (Frank 2005). Research shows that cognitive and non-cognitive performance are positively correlated (Eva et al. 2009), although a recent study by Lucieer et al. (2015) in this journal indicates that “the use of only non-cognitive selection criteria is not sufficient to select the best academically performing students.” We have not found studies comparing separate cognitive and non-cognitive procedures within one cohort in one medical school. Further research is needed to explore the predictive validity of non-cognitive methods compared to a method like ours in one medical school.

## Conclusion

Our curriculum sample selection procedure does explain performance in medical school. It adds to secondary school cognitive performance (pu-GPA). It is attractive for its efficiency. All those interested to apply for medical school can participate because large groups can be tested simultaneously, eliminating the need to preselect applicants. Our procedure may be especially useful in countries that are unable to take pu-GPA reliably into account in selection procedures.
